# The effect of a new communication template on anticipated willingness to initiate or resume allergen immunotherapy: an internet-based patient survey

**DOI:** 10.1186/s13223-015-0083-z

**Published:** 2015-05-22

**Authors:** Moises A. Calderon, Linda Cox, Thomas B. Casale, Ralph Mösges, Oliver Pfaar, Hans-Jørgen Malling, Joaquin Sastre, Musa Khaitov, Pascal Demoly

**Affiliations:** Section of Allergy and Clinical Immunology, Imperial College London - National Heart & Lung Institute, Royal Brompton Hospital, Dovehouse Street, London, United Kingdom; Nova Southeastern University, Davie, FL USA; Internal Medicine, Morsani College of Medicine, University of South Florida, Tampa, FL USA; Universitätsklinikum Köln, Universität zu Köln, Köln, Germany; Center for Rhinology and Allergology, Wiesbaden, Germany; Department of Otorhinolaryngology, Head and Neck Surgery, Universitätsmedizin Mannheim, Medical Faculty Mannheim, Heidelberg University, Mannheim, Germany; Allergy Clinic, Danish AllergyCenter, Dermato-Allergological Department, Gentofte University Hospital, Copenhagen, Denmark; Allergy Department, Fundación Jiménez Díaz, Madrid, Spain; NRC Institute of Immunology FMBA, Moscow, Russian Federation; Department of Pulmonology - Division of Allergy, Hôpital Arnaud de Villeneuve, University Hospital of Montpellier, Montpellier, France; Sorbonne Universités, UPMC Paris 06, UMR-S 1136, IPLESP, Equipe EPAR, Paris, France

**Keywords:** Allergen immunotherapy, Allergy, Adherence, Information, Patient-physician communication

## Abstract

**Background:**

A patient’s knowledge of his/her allergic condition and treatment is a key factor in adherence and effectiveness.

**Methods:**

To assess patients’ understanding of allergy and acceptance of allergen immunotherapy on the basis of (i) information given by their physician at the time of prescription and (ii) a new communication template viewed some months later, we performed an Internet-based survey of patient panels in France, Germany, Spain, the USA and Russia. The survey participants were either recent “early abandoners” (having discontinued allergen immunotherapy before the end of the prescribed course) or “non-starters” (having decided not to initiate a course of allergen immunotherapy recommended by their physician). All participants completed an on-line questionnaire immediately before and immediately after viewing the new communication template. The study’s main objectives were to validate the new communication template and to assess its impact on anticipated willingness to initiate or resume allergen immunotherapy.

**Results:**

We surveyed a total of 261 patients (France: 57; Germany: 51; Spain: 52; USA: 51; Russia: 50), comprising 127 “early abandoners” and 134 “non-starters”. The mean time since symptom onset and selection for the study was 14.5 years. Subcutaneous allergen immunotherapy had been prescribed in 60 % of cases. Twenty-eight percent of the participants did not know for which allergy they were being treated. Early abandoners reported a perception of low effectiveness (39 %) and complained about expense (39 %) and practical constraints (32 %). Twenty-two percent of the non-starters feared side effects. The communication template was considered to be clear (by 92 % of the patients), convincing (by 75 %) and reassuring (by 89 %); 80 % of the participants felt better informed afterwards, and 67 % stated that viewing the communication template would have made them more likely to continue or initiate allergen immunotherapy (overall willingness score: 5.65 out of 10 before viewing and 7.1 out of 10 afterwards).

**Conclusions:**

After viewing a new communication template on allergy and allergen immunotherapy, patients participating in the survey felt better informed and more likely to initiate or complete this therapy. It now remains to investigate the communication template’s effect on actual acceptance of and adherence to allergen immunotherapy.

**Electronic supplementary material:**

The online version of this article (doi:10.1186/s13223-015-0083-z) contains supplementary material, which is available to authorized users.

## Background

Allergic rhinitis (AR) is a chronic, immunoglobulin E (IgE)-mediated inflammatory disease of the upper airways that affects a signification proportion of adult and paediatric populations worldwide [1–3]. According to a recent review, the patient-reported prevalence of the symptoms of AR is 7.2–54.1 % in Africa, 5.5–45.1 % in Latin America, 12–30 % in North America, 23–30 % in Europe and 18.2 % in the Russian Federation [4, 5], although physician-diagnosed prevalences are lower. Furthermore, AR is a known risk factor for the development of allergic asthma [6]. The condition has a considerable socioeconomic burden and impairs health-related quality of life [7, 8]. Although most cases of AR are treated with symptomatic drugs such as H1-histamine receptor antagonists and/or nasal corticosteroids [1–3], about 20 % of patients do not achieve disease control [9].

According to most guidelines, allergen immunotherapy (AIT) is a treatment option for patients with moderate-to-severe IgE-mediated respiratory allergies and in whom medications such as antihistamines and moderate-dose topical glucocorticoids provide insufficient symptom control [1, 2, 10–14]. AIT is an effective, disease-modifying treatment for AR [15–18]. Furthermore, a recent review found that 23 out of 24 health economics studies of AIT compellingly demonstrated the cost savings conferred by AIT over symptomatic drug treatment [19].

Although AIT starts to act on symptoms within a few weeks or months [20–22], courses of several years are needed to switch the immune system to a tolerogenic regulatory T cell profile and Th2 to Th1 immune rebalancing [1, 2, 13, 16, 17]. Good adherence to AIT is essential for effectiveness and comprises a series of components: initiation, implementation (also referred to as compliance) and discontinuation (the reciprocal of which is persistence). Adherence depends on several interacting factors, including inconvenience, cost factors, treatment benefit (or lack of, and as perceived by both patients and physicians) and the patient’s level of knowledge about his/her allergic condition and treatment [23–29]. In the literature, the implementation/compliance component of adherence to AIT (defined as the percentage of prescribed medication actually taken by the patient) ranges from around 25 % to over 90 %. These values come from studies that vary in terms of the patient population, allergen preparation, the administration route (subcutaneous or sublingual), the administration regimen (continuous or discontinuous), the frequency of administration and the overall treatment period (from several months to several years) [23, 27–29]. Nevertheless, most reported values are between 60 % and 80 %. In terms of the discontinuation component of AIT, Anolik et al.’s retrospective health record review found that 69 % of 231 patients having discontinued AIT before the end of the three-year treatment period did not have a specific, physician-recorded, reason for discontinuation (despite regular consultations during this period) or did not return to the office for further evaluation or treatment [30]. In a retrospective “real-life” review of a pharmacy database containing data on 6486 patients having initiated AIT with one or more of the allergens of interest between 1994 and 2009, Kiel et al. found that a specialist prescriber, single-allergen AIT, lower socioeconomic status and younger age were all independent predictors of premature discontinuation (i.e. poor persistence) [31]. One can speculate that use of single-allergen AIT to treat potentially polysensitized patients may have had a negative impact on adherence in Kiel et al.’s study. Overall, these results suggest that a factor broadly defined as “motivation” is important in adherence in general and persistence in particular.

The objective of the present international, observational, Internet-based survey was to assess patients’ understanding of and commitment to AIT based on (i) the information provided during consultation with the prescribing physician and then (ii) following presentation of a new communication template about allergy and AIT. We focused solely on “non-starters” (i.e. patients who had elected not to initiate AIT, despite their physician’s recommendation), and “early abandoners” (i.e. patients who had prematurely ceased a prescribed course of AIT). Furthermore, we measured the impact of a new communication template on the survey participants’ willingness to start or resume treatment.

## Methods

### Survey design and population

We performed an observational, Internet-based survey in five countries around the world (France, Germany, Spain, the USA and Russia). These countries were selected to reflect markets in which AIT is well established (France, Spain, USA and Germany) and/or is likely to develop even further (Russia and the USA, where SLIT is currently being launched). In each country, we contacted members of patient panels previously constituted by market research organizations and invited them to participate in an Internet-based survey. In view of the observational, non-interventional nature of this Internet-based survey, specific ethical and regulatory approval was not required. The study therefore does not have a clinical trial number. The panel members had provided their general consent to participation in opinion surveys and subsequent exploitation of the collected data but had not provided individual consent to this survey. The participants were not necessarily being treated with AIT products from the study sponsors (Stallergenes SA, Antony, France, and ALK-Abelló A/S, Hørsholm Denmark). Participants screened themselves for eligibility (i.e. through self-reporting) with a short Internet questionnaire and were not examined by a physician as part of the selection process. The inclusion criteria were as follows: (i) age 18 or over, (ii) physician-diagnosed AR (regardless of the inducing allergen or time since diagnosis); (iii) moderate-to-severe nasal and ocular symptoms (self-reported); (iv) allergic symptoms for at least one month per year (self-reported); (v) a recommendation of AIT by the participant’s physician (whether a specialist or a primary care physician) within the previous 12 months; (vi) recollection of the information about allergy and AIT given by the prescribing physician; and (vii) discontinuation of AIT before the end of the recommended course (for “early abandoners”) or refusal to start a course of AIT (for “non-starters”). Approximately equal numbers of early abandoners and non-starters were recruited. Individuals intending to start AIT in the coming weeks or taking AIT at the time of the screening step were excluded from the survey. Lastly, people who had been told to discontinue AIT by a physician were excluded from the survey.

### The survey questionnaire

Eligible participants filled out a 41-item, Internet-based questionnaire (developed by the panel of authors specifically for this survey: see the Additional file 1) in March and April 2014. The questionnaire was translated into local languages from English and then translated back into English for validation and cultural adaptation [32]. The questionnaire covered (i) the participant’s allergies, symptoms and treatments, (ii) the participant’s perception of the information on allergy and AIT provided by the physician at the time AIT was recommended, (iii) the participant’s perception of the new communication template on allergy and AIT (see below), and (iv) willingness to start or resume AIT after having viewed a new communication template (see below). All questionnaire data were anonymous. No directly or indirectly nominative information was recorded. Some of the questions were open-ended. The Internet-based survey questionnaire took around 15 min to fill out.

### The new communication template

The new communication template on allergy and AIT (Table 1) was developed (in English) in several rounds by drawing on the authors’ personal experience and the scientific literature on the types and formats of health information preferred by patients and physicians [33]. The goal was to summarize information on the characteristics of allergic disease (in one section) and the features and mode of action of AIT (in another section) in concise, lay terms. Any disagreements were resolved by consensus. A draft template was tested in four countries (France, Germany, Russia and the USA) in the local language by presentation to a total of 445 physicians (including general practitioners, allergy specialists and both AIT prescribers and non-prescribers), pharmacists, and patients with respiratory allergies. The draft was then modified according to the feedback received. In the present study, the new communication template was presented to the survey participants immediately after they had completed the study questionnaire. Immediately after presentation of the new communication template, the study questionnaire was then administered again, in order to measure any changes in views and opinions.

**Table 1:** The new communication template on allergy and AIT

About respiratory allergies
• Respiratory allergy results from a disorder of the immune system
• Respiratory allergy is a chronic disease caused by both a genetic predisposition and environmental factors. In predisposed persons, exposure to several factors (such as pollution, smoking, and climate change) can cause or exacerbate allergy.
•Respiratory allergy is a progressive disease that gradually worsens over time, with an increased risk of polysensitization and asthma
• Respiratory allergy has severe consequences:
- Direct disease burden: symptoms impair everyday activities and degrade the quality of sleep, inducing fatigue and impacting learning and attention.
- Impact on work/school performances: among chronic diseases, allergic rhinitis has the highest impact on productivity.
About allergy immunotherapy
Main definition of AIT:
Allergy immunotherapy (AIT) is the only allergy treatment with a long-lasting effect on all symptoms.
AIT induces tolerance to allergens by rebalancing the immune system.
AIT is a targeted and efficient solution.
Key messages
• Which patients is AIT for?
- AIT is mainly dedicated to patients in whom symptomatic medications are insufficiently effective or poorly tolerated.
• How does it work?
- AIT is a targeted solution: After an accurate diagnosis, patients receive a tailored treatment.
- AIT is a disease-modifying allergy treatment that acts on the immune system itself by rebalancing it (in contrast to symptomatic drugs like antihistamines and corticoids, which only temporarily stop the symptoms of allergic reactions).
- There are several mode of administration: injections to be given monthly at the medical office or drops placed under the tongue, according to the preferences and needs of the patient. Tablets are available for grass pollen allergies.
• AIT is an efficient solution:
- Only one treatment active on all symptoms (in contrast to most of the antihistamines and corticoids that affect primarily nasal or eye symptoms).
- Efficacious over the long-term: Efficacy is sustained over successive years even after the treatment is stopped
- Reduces the use of symptomatic medication (antihistamines and/or corticoids).

### *Data management*

A descriptive analysis of the survey data was performed with SPSS software (version 15.0.1, IBM Corporation, Armonk, USA). Quantitative parameters are expressed as the mean, and qualitative parameters are expressed at a percentage of the corresponding survey population or subpopulation.

## Results

### Characteristics of the survey population (Table 2)

**Table 2:** Characteristics of the survey population

	France	Germany	Spain	USA	Russia	All countries
Number of patients (M/F):						
total population	57 (18/39)	51 (20/31)	52 (31/21)	51 (16/35)	50 (15/35)	261 (100/161)
*“early abandoners”*	*25 (11/14)*	*25 (15/10)*	*26 (19/7)*	*26 (8/18)*	*25 (9/16)*	*134 (62/72)*
*“non-starters”*	*32 (7/25)*	*26 (5/21)*	*26 (12/14)*	*25 (8/17)*	*25 (6/19)*	*127 (38/89)*
Mean [range] age (years):						
total population	41 [19–72]	40.8[18–70]	35.3[22–61]	42 [22–70]	37.0 [18–60]	39.4 [18–72]
*“early abandoners”*	37.9 [19–72]	35.5 [18–67]	35.3 [23–59]	39.5[26–58]	37.4 [18–60]	37.1 [18–72]
*“non-starters”*	43.2 [21–63]	46 [20–70]	35.4 [22–61]	44.5 [22–70]	36.7 [18–53]	41.4 [18–70]
Mean [range] time since allergy						
onset (years):						14.5
total population	11.0 [1–33]	12.0 [1–45]	13.5 [1–40]	18.8 [2–64]	12.5[2–33]	[1–64].
*“early abandoners”*	9.9 [2–30]	8.3 [1–45]	11.4 [1–30]	17.4 [2–54]	10.7 [2–30]	11.4 [1–54]
*“non-starters”*	11.9 [1–33]	15.6 [1–44]	15.5 [1–40]	20.3 [2–64]	14.2 [3–33]	15.3 [1–64]
Stated allergies:						
grass pollen	78 %	84 %	58 %	96 %	68 %	77 %
house dust mite	53 %	59 %	65 %	78 %	36 %	58 %
tree pollen	67 %	45 %	52 %	90 %	40 %	59 %
animal dander	40 %	35 %	46 %	67 %	38 %	45 %
other	3 %	8 %	12 %	29 %	8 %	12 %
Moderate-to-severe impact of						
allergy on personal life:						
total population	79 %	77 %	81 %	96 %	96 %	85 %
*“early abandoners”*	82 %	65 %	81 %	92 %	96 %	83 %
*“non-starters”*	76 %	88 %	81 %	100 %	96 %	88 %
Moderate-to-severe impact of						
allergy on professional life						
total population	65 %	63 %	70 %	76 %	88 %	73 %
*“early abandoners”*	60 %	38 %	77 %	76 %	84 %	71 %
*“non-starters”*	69 %	88 %	62 %	77 %	92 %	75 %
Patient consulting a specialist as						
their main physician:						
total population	54 %	69 %	68 %	44 %	62 %	59 %
*“early abandoners”*	36 %	54 %	58 %	30 %	60 %	45 %
*“non-starters”*	68 %	84 %	77 %	58 %	64 %	74 %

**Fig. 1:**
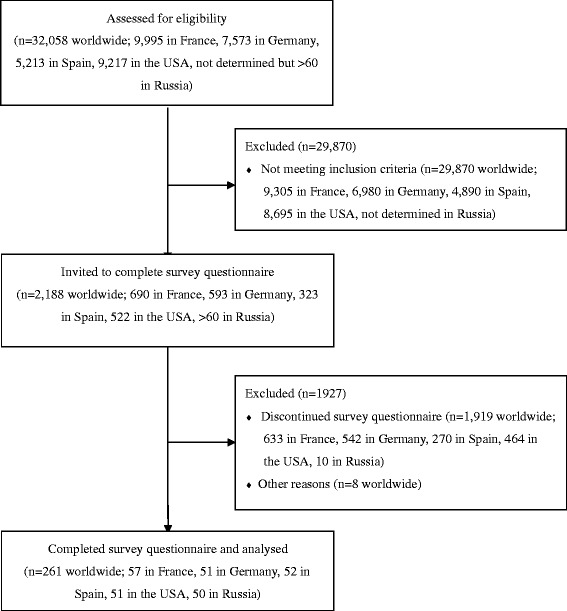
The study flow chart

A total of 261 eligible participants (France: n = 57; Germany: n = 51; Spain: n = 52; USA: n = 51; Russia: n = 50) were included in the Internet-based survey and filled out the survey questionnaire (Fig. 1). For the population as a whole, the mean time between the onset of allergic symptoms and inclusion in the survey was 14.5 years. During this time, the participants had been treated with a range of symptomatic medications and (for early abandoners) AIT. There were 134 non-starters (France: n = 32; Germany: n = 26; Spain: n = 26; USA: n = 25; Russia: n = 25) and 127 early abandoners (France: n = 25; Germany: n = 25; Spain: n = 26; USA: n = 26; Russia: n = 25). The non-starters were more likely to be consulting a primary care physician (relative to early abandoners).

Unfortunately, precise data on the duration of AIT in early abandoners were not available. However, the great majority (79 %) of the early abandoners had discontinued AIT after several months (rather than a few days). The proportion of early abandoners having discontinued AIT after several months (rather than a few days) differed markedly from one country to another (France: 60 %; Germany: 100 %; Spain: 81 %; USA: 85 %; Russia: 68 %). Although data on allergen sensitization were not available, the triggering allergens most frequently reported by the participants were grass pollen (78 %), tree pollen (67 %), house dust mites (53 %) and animal dander (40 %) (Table 2).

The proportion of participants reporting that AR had a moderate-to-severe impact on their personal life ranged from 76 % to 100 %, depending on the country and the subgroup (Table 2). The corresponding values for the impact on professional life were only slightly lower (38 % to 92 %; Table 2). Concomitant asthma (though not necessarily allergic) was reported by 27 % of the participants. Non-starters were more likely than early abandoners to report that their main physician in the treatment of their allergy was a primary care physician (rather than a specialist).

### The prescribed course of AIT and reasons for non-initiation or discontinuation of treatment (Table 3)

**Table 3:** Characteristics of the prescribed AIT preparations and administration regimens

	France	Germany	Spain	USA	Russia	Overall study population
Proportion of patients unaware of the type of allergen being administered:						
total population	46 %	18 %	29 %	20 %	24 %	28 %
*“early abandoners”*	40 %	8 %	19 %	23 %	12 %	20 %
*“non-starters”*	50 %	27 %	38 %	16 %	36 %	34 %
Type of AIT formulation prescribed in the overall population:						
*SCIT*	38 %	61 %	33 %	88 %	82 %	60 %
*SLIT drops*	31 %	10 %	21 %	8 %	4 %	15 %
*SLIT tablets*	24 %	27 %	46 %	4 %	14 %	23 %
*unspecified*	7 %	2 %	0 %	0 %	0 %	2 %
Proportion of patients prescribed with a continuous regimen	38 %	27 %	38 %	76 %	28 %	41 %

Overall, SCIT had been prescribed in 60 % of cases (in 57 % of early abandoners and 62 % of non-starters). The proportion of participants prescribed with SCIT was higher in Germany, the USA and Russia (over 60 %) than in France and Spain (below 40 %) (Table 3). Overall, 41 % of the administration regimens were continuous (i.e. year-round), 53 % were discontinuous (e.g. pre- and co-seasonal or co-seasonal only, for intermittent allergies) and 5 % were not specified.

Strikingly, 28 % of the survey participants did not know with which allergen they were being treated (France: 46 %; Germany: 18 %; Spain: 29 %; USA: 20 %; Russia: 24 %). Unsurprisingly, this percentage was higher in non-starters (34 %) than in early abandoners (20 %), who had actually received the treatment to some extent.

Among the early abandoners, the main reasons for stopping AIT were insufficient perceived efficacy (39 % on average, ranging from 16 % in France to 68 % in Germany), financial expense (39 %), practical constraints (32 % on average, ranging from 16 % in Germany to 56 % in France), no perceived change in symptoms (25 %), and receipt of discouraging information about AIT (9 %, primarily from relatives and the media). Among the non-starters, the main reasons for not starting were financial expense (34 %), practical constraints (31 %), insufficient perceived benefits (25 %) and fear of adverse events (22 %).

### The participant’s perception of the information on allergy and AIT provided by the physician

Overall, 27 % of the participants had not been told that respiratory allergy was a chronic condition, 36 % had not been told that respiratory allergy was an immune disorder and 24 % had been told that respiratory allergies can worsen. A small but non-negligible proportion of participants (7 %) had not been told about any of these aspects (with as many as 16 % in the USA).

Although 91 % of participants had been told how AIT is thought to work, only 34 % could recall being told about the overall duration of AIT (2.7 years, on average, according to the participants who recollected hearing this information) and only 66 % could recall being informed about safety (ranging from 45 % in France to 80 % in the USA and in Germany). Thirty-three percent of the participants (ranging from 22 % in Germany to 40 % in France to) stated that they were not asked about their treatment preferences.

When asked to spontaneously recall the potential benefits of AIT as presented by their physician, 26 % mentioned nasal and ocular symptom relief, 22 % reported a long-term solution, 16 % reported an improvement in quality of life and 5 % mentioned a reduction in medication use. However, when prompted, 76 % of the participants reported that the physician did indeed mention symptom relief, with 51 % for a long-term solution, 72 % for an improvement in quality of life, and 52 % for a reduction in medication use.

When asked to spontaneously recall the potential disadvantages of AIT, 22 % mentioned the overall duration of treatment, 12 % mentioned lack of perceived efficacy, and 8 % mentioned adverse events. Strikingly, 14 % of the participants reported that they did not spontaneously recall any disadvantages being mentioned. According to the participants, only 34 % of the physicians mentioned the total treatment duration.

The participants had been asked to state their willingness to undergo AIT on the basis of the physician’s presentation of allergy and AIT. On a numeric scale from 0 (least willing) to 10 (most willing), the mean score for the overall survey population was 5.7 (early abandoners: 6.2; non-starters: 5.1). There were slight differences in the mean score from one country to another for early abandoners (France: 6.4; Germany: 5.6; Spain: 7.6; USA: 6.2; Russia: 5.0) and for non-starters (France: 4.7; Germany: 4.9; Spain: 6.7; USA: 4.4; Russia: 5.0).

### The participant’s perception of the new communication template on allergy and AIT

**Fig. 2:**
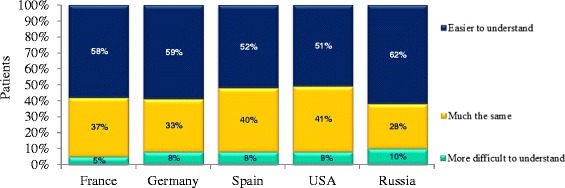
Understanding of the new communication template. In each country, participants were asked “How does this new presentation of AIT [the new communication template] compare with that given by your physician?”. The possible answers were “Easier to understand”, “More difficult to understand” and “Much the same?”

Participants considered the new communication template on allergy and AIT (Table 3) to be clear (92 %), convincing (75 %) and reassuring (89 %), and they felt better informed as a result (80 %). The new communication template was considered to be more detailed (65 %), easier to understand (56 %) and more convincing (65 %) than the information that participants had received in their physician’s office (Fig. 2). Only 8 % of the participants felt that information about AIT given by the physician was absent from the communication template. In contrast, 57 % considered that information given by the communication template was not included in the physician’s presentation. Ninety-two percent of the participants considered that the way AIT works was clearly explained in the communication template.

After viewing the parts of the new communication template on allergy, 57 % of the participants spontaneously recalled that allergy is an immune disorder. Few participants (4 %) participants spontaneously recalled that AR can progress to asthma. prompted by the on-screen appearance of closed “yes”/“no” questions on this topic, 84 % of the participants stated that allergy is an immune disorder, and 72 % stated that AR can progress to asthma. After viewing the parts of the communication template on AIT, a minority of participants spontaneously recalled that AIT can improve quality of life (31 %), that AIT is effective over the long term (29 %) and that there are several possible administration routes (30 %). However, when subsequently prompted with closed “yes”/“no” questions on this topic, the majority of the participants stated that AIT has long-term efficacy (72 %), can reduce medication use (70 %), relieve both nasal and ocular symptoms (65 %), “rebalances” the immune system (54 %) and can be administered by several different routes (51 %).

**Fig. 3:**
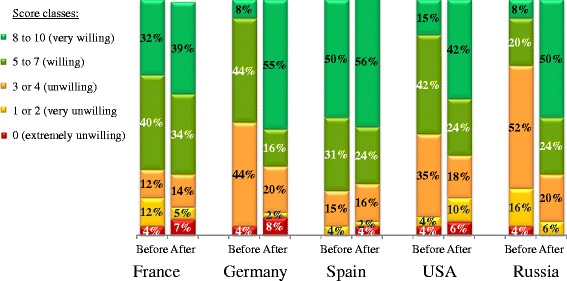
Willingness to initiate or resume AIT. In each country, participants were asked to state their willingness to initiate or resume AIT before and then after presentation of the new information template (from 0, least willing, to 10, most willing). The results are presented by score class

After having viewed the communication template, 89 % of the participants stated that AIT might meet their needs, and 76 % of participants stated that they would have been more likely to start or continue an AIT treatment if their physician had used the new communication template. Furthermore, 68 % of participants stated that they were currently willing to start/resume AIT treatment. When asked to quantify their willingness to start or resume a course of AIT, the mean score was 7.1 out of 10 (i.e. an increase of 1.5 points over the score rated before presentation of the communication template). To examine the participants’ “before vs. after” data in more detail, we grouped the individual scores into classes: a score of 8 to 10 was defined as “very willing”, with 5 to 7 defined as “willing”, 3 or 4 as “unwilling”, 1 or 2 as “very unwilling” and 0 as “extremely unwilling” (Fig. 3). The proportion of participants who were either “willing” or “very willing” to start or resume a course of AIT increased markedly after viewing the communication template in all of the countries other than Spain (Fig. 3). The most marked increase in willingness was recorded in Russia, where the proportion of participants either “willing” or “very willing” to start or resume a course of AIT was 28 % before viewing the communication template and 74 % afterwards.

## Discussion

The quantifiable components of medication adherence (initiation, implementation, and discontinuation/persistence) are variously affected by many interacting factors, including the patient’s preferences and views on treatments and personal needs, adequate knowledge of the disease, perceived health risks, costs, health literacy, concerns about disease worsening and complications, expectations about treatment outcomes and concerns about side effects [23–31].

In the present multinational, Internet-based survey, we focused on the participant’s knowledge of their respiratory allergic disease and AIT and the relationship between this knowledge and willingness to start or resume this type of therapy. The survey was designed to assess participants’ understanding of allergy and their acceptance of allergen immunotherapy on the basis of information provided by their physician at the time of prescription and information in a new communication template viewed some months later. In contrast, the survey was not designed to (i) compare “early abandoners” with “non-starters”, (ii) compare dissatisfied participants with satisfied participants or, more generally, (iii) determine which factors may be associated with unwillingness to initiate or continue AIT.

The survey population comprised patients with moderate to severe symptoms and who had been suffering from allergies for many years (an average of 14.5 years prior to inclusion in the survey). The results of the present Internet-based survey show clearly that the participants were either not optimally informed about allergic disease and AIT by their physician, or had forgotten some of the key information given since their consultation with the physician. This lack of knowledge was most worryingly evidenced by the fact that 28 % of the participants did not know with which allergen they were being treated, which can influence the perception of efficacy according to some allergens. Furthermore, 27 % of participants did not know that allergy is a chronic disease and 24 % were unaware of the progressive nature of allergy. Hence, lack of knowledge may have prompted the “early abandoners” to discontinue AIT. The main reasons for discontinuation by the “early abandoners” were lack of perceived efficacy (39 %) and cost (39 %). In a review on adherence to AIT, Senna et al. considered that the main reasons for discontinuation (i.e. lack of persistence) were inconvenience, a lack of efficacy, costs and loss of working hours, and side effects [23]. In studies of real-life practice with infrequent consultations, lack of perceived efficacy may be an important driver of poor adherence. Although AIT starts to relieve symptoms within a few weeks or months [20–22], patients may have unrealistic expectations of quick improvements in symptoms (such as those they may have experienced with antihistamines and nasal corticosteroids). We suggest that patients’ adherence to AIT could be enhanced by more accurate presentation of this treatment modality to patients, making them better prepared for progressive (and thus less immediately visible) changes. This issue is also addressed in the recently published guideline on AIT by the German, Austrian and Swiss allergic societies, which provides a ‘treatment information sheet’ that informs the patient about practical aspects of AIT (such as expected effects, the type and duration of treatment, possible side effects and alternative treatments) [34]

Conversely, the high levels of adherence found in clinical trials result (in part) from regular monitoring of the enrolled patients [28], and so non-adherence in these trials depends more on the patient’s monitoring and motivation than on his/her perception of inefficacy or other causes. Hence, as in clinical trials, a solid partnership between the patient and the physician throughout the course of AIT is necessary for treatment success routine clinical practice. We suggest that the physician should monitor changes over time in efficacy and outcomes, in order to improve levels of dialogue and boost the patient’s motivation.

The present survey had a number of limitations, many of which are inherent to observational, self-reported, Internet-based surveys. The small sample sizes prevented statistical analysis, and so assessment of any significant differences in profile between “non-starters” and “early abandoners” was not possible. The small number of participants in each country and lack of knowledge of clinical profiles prevented us from applying statistical tests and comparing one country with another. Given that participants had to fill out a screening questionnaire to check that the selection criteria were met, it may be that only highly motivated participants completed this stage and thus did not represent a typical patient population (i.e. selection bias). The data were anonymous and self-reported over the Internet, which may have introduced bias. Some of the clinical details (such as the nature of the allergy-inducing allergens in a given participant) may not have been recently validated by a physician. The participants were not necessarily AIT-naïve, and may have initiated (and perhaps completed) a course of AIT previously. However, all participants considered for eligibility had physician-diagnosed AR. Furthermore, the participants’ self-assessments of symptom severity and impact on quality of life were not recorded with standardized, validated tools. The survey may have suffered from recall bias (especially concerning the information presented by the physician), since the participants were questioned up to 12 months after the consultation in which AIT was recommended. It is possible that participants had been well informed at the consultation but had since forgotten part of the information; this may have artificially improved the participants’ perception of the new communication template. The “baseline” levels of information about AIT and allergy doubtless vary from one country to another and from one physician or specialty to another. Furthermore, there were doubtless marked differences in the extent to which and the way in which the physicians provided the participants with information (i.e. conversation, leaflets, Internet documents, etc.). In the US, for example, patients receiving SCIT at a health care facility other than the prescribing office read and approve a detailed information and consent letter [35]. In contrast, the new communication template was always viewed on the screen of a computer or other Internet-connected device. Furthermore, the study questionnaire was developed by the authors by consensus and was not extensively tested or psychometrically validated prior to use in the study. Lastly, we assessed acceptance of and motivation for AIT, rather than quantifiable components of adherence *per se*.

In the present Internet-based survey, viewing of a communication template was associated with markedly better recall of the features of allergy and AIT when the survey participants were prompted by the on-screen appearance of closed “yes”/“no” questions on a given topic. In contrast, spontaneous recall of these features was suboptimal. In clinical practice, however, the communication template would be presented and discussed with the physician, and so better spontaneous recall could be expected in an interactive context. Use of the present communication template revealed the need for (and value of) simple, objective, defined information on allergy and AIT.

## Conclusion

For optimal clinical effectiveness, a course of AIT should be completed as part of a mutually agreed “moral contract” between the patient and his/her physician, where both partners “buy in” to the disease management strategy and each has duties and obligations in maintaining or improving health. The way in which the physician presents AIT to his/her patient is crucial in (i) providing accurate information for decision-making and (ii) increasing levels of commitment and adherence to therapy.

We found that after consulting the physician treating their allergy, many survey participants did not receive or retain important information about their allergic disease and AIT—including items that would probably have increased their degree of willingness to initiate or continue AIT. After having viewed a new information template on line, survey participants with allergies felt better informed and stated that they were more likely to initiate or resume AIT. However, the present survey’s limitations (a small sample size, a lack of detailed knowledge of clinical profiles, possible selection bias, self-reporting of allergy status, and a lack of standardized assessments of symptom severity and quality of life) mean that the relationship between the new information template and actual adherence to AIT in clinical practice must be tested in a controlled study.

## Additional file

Additional file 1:
**The survey questionnaire.**

## Abbreviations

AIT: Allergen immunotherapy
AR: Allergic rhinitis
IgE: Immunoglobulin E
SCIT: Subcutaneous allergen immunotherapy
SLIT: Sublingual allergen immunotherapy
