# Supplementary material for: The effect of a new communication template on anticipated willingness to initiate or resume allergen immunotherapy: an internet-based patient survey
Source: Allergy Asthma Clin Immunol. 2015 May 22;11(1):17. doi: 10.1186/s13223-015-0083-z (PMC4443522; doi:10.1186/s13223-015-0083-z)
Supplement: Additional file 1: — The survey questionnaire. [file 13223_2015_83_MOESM1_ESM.docx]

Additional file. The survey questionnaire.

**Preliminary questions**

1. Which of the following airborne allergens are you allergic to?
   *You may give several answers, if applicable*

- Grass pollens
- Tree pollens
- House dust mites
- Animal hair/dander
- Other aeroallergens (please specify):_____________________

1. Which of the following nasal, eye, chest and other symptoms (i.e. allergic rhinitis with or without conjunctivitis) do you suffer from?

*You may give several answers, if applicable*

- Runny nose
- Blocked nose
- Itchy nose
- Sneezing
- Itchy eyes
- Tearing
- Red eyes
- Swollen eyes
- Difficulty breathing
- Wheezing
- Cough
- Asthma
- Itchy throat
- Sore throat
- Itchy mouth
- Itchy ears
- Eczema
- Skin rash
- Other symptoms (please specify):_____________________

1. How would you evaluate the impact of these symptoms on your personal life?
   *Please give one answer only*

- No impact
- A slight impact
- A moderate impact
- A severe impact

1. How would you evaluate the impact of these symptoms on your professional life?
   *Please give one answer only*

- No impact
- A slight impact
- A moderate impact
- A severe impact

1. Who is the key physician (the one you consult most) for the management of your allergies?
   *Please give one answer only*

- General practitioner/family physician
- Allergist
- Pulmonologist
- ENT
- Dermatologist
- Pediatrician
- Other (please specify):_____________________

1. What type of medicines do you take for your allergies?
   *You may give several answers, if applicable*

- Antihistamines
- Nasal sprays/drops
- Eye drops
- Corticoids/corticosteroids
- Asthma drugs
- Homeopathic medicines
- Other (please specify):_____________________

1. What is satisfactory about those medicines?

*Open-ended question*

1. What is unsatisfactory about those medicines?

*Open-ended question*

1. What are your expectations in terms of your allergy treatment?

*Open-ended question*

1. What would you need to improve the management of your allergy?

*Open-ended question*

**Section 1
Recall/perception of the information about allergen immunotherapy (AIT) provided by the physician at the time of consultation**

1. Which words would you use to describe the presentation of allergen immunotherapy (AIT, also known as desensitization) given by your physician?

*Open-ended question*

1. Did a physician explain to you that respiratory allergy…
   1. Is a chronic disease?

- Yes
- No
  1. Results from a disorder of your immune system?
- Yes
- No
  1. Is associated with a risk of worsening (i.e. the risk of developing an allergy to other allergens and/or developing asthma over time)?
- Yes
- No

1. Do you know which allergen(s) was/were targeted by the AIT your physician recommended or prescribed?

- Yes

If yes, please specify: *You may give several answers, if applicable*

- - Grass pollens
  - Tree pollens
  - House dust mites
  - Animal hair/dander
  - Other (please specify):____________________
- No

1. In terms of the AIT:
   1. Did the physician explain to you how the treatment works?

- Yes
- No
  1. Did the physician speak about possible side effects?
- Yes
- No
  1. Did the physician asked you about your treatment preferences / expectations before prescribing it to you?
- Yes
- No
  1. What form of AIT did the physician propose?
     *Please give one answer only*
- Injections
- Drops
- Tablets
- Don’t know
  1. What was the treatment period suggested by the physician:
     *Please give one answer only*
- All year long
- Discontinuous (only a part of the year)
- I can't remember
  1. Did the physician mention the total duration of the treatment (e.g. for how many years you should take the treatment)?
- Yes
- No

If Yes: what total duration of treatment was mentioned?

*Please enter a number between 1 and 30*

/_____/ years

1. What were the most convincing arguments about the presentation/proposal of AIT?

*Open-ended question*

1. And what were the least convincing arguments about the presentation/proposal of AIT?

*Open-ended question*

1. Which aspects of AIT did you perceive to be barriers to treatment?

*Open-ended question*

1. Which benefits of AIT did the physician talk about?

*You may give several answers, if applicable*

- Better quality of life
- Reduction in nose/eye symptoms
- Reduction in the use of symptomatic medications (antihistamines, corticoids, etc.)
- An overall, specific treatment for allergy
- Complete relief of symptoms
- Efficacy over the long term or a cure for the allergy
- Efficacy visible in the first few months
- A preventive effect on other allergies and on asthma
- Other (please specify):_____________________

**For "early abandoners" only (*i.e. patients who started the treatment but stopped it early)***

1. How long did you take the AIT for?

*Please give one answer only*

- Several days
- Several months

1. Which expectations prompted you agree to the prescription of AIT?

*You may give several answers, if applicable*

- To reduction the symptoms within weeks/years
- To get rid of nose/eye symptoms
- To limit the use of symptomatic medications
- To cure your allergy in the long term
- To prevent the development of other allergies and/or asthma
- To improve your quality of life
- Because you trust your physician
- Because AIT was recommended by relatives
- Other (please specify):_____________________

1. Could you explain why you stopped your AIT prematurely?

*You may give several answers, if applicable*

- The treatment was too burdensome (please specify):
  - The need to go to the physician's office frequently was too demanding.
  - The requirement to take the AIT once a day or several times a week was too frequent.
  - The total treatment duration was too long.
- The treatment was too expensive
- You received some discouraging information about AIT from:
  - The internet
  - The media
  - Relatives
  - Your general practitioner/family doctor
  - Another physician (please specify): ………
- You perceived the treatment to be weakly effective or ineffective.
- You did not perceive any change in your symptoms.
- Other (please specify):_____________________

1. Today, how would you evaluate your willingness to start or resume AIT, on a scale from 0 (you absolutely do not want to start or resume AIT) to 10 (you absolutely want to start or resume AIT)?

*Please enter a number between 0 and 10*

/_____/

**Non-starters only (*i.e. patients who did not start AIT)***

1. Why didn't you start the AIT after the physician had presented/suggested it?

- The treatment was too burdensome (please specify):
  - The need to go to the physician's office frequently was too demanding.
  - The requirement to take the AIT once a day or several times a week was too frequent.
  - The total treatment duration was too long.
- The treatment was too expensive
- Your symptoms were not severe enough
- You received some discouraging information about AIT from:
  - The internet
  - The media
  - Relatives
  - Your general practitioner/family doctor
  - Another physician (please specify): ………
- You were not convinced by the physician's presentation
- You were afraid of possible side effects
- You did not perceive AIT as having benefits over symptomatic drugs (antihistamines, corticoids, etc.)
- Other (please specify):_____________________

1. Today, how would you evaluate your willingness to start or resume AIT, on a scale from 0 (you absolutely do not want to start or resume AIT) to 10 (you absolutely want to start or resume AIT)?

*Please enter a number between 0 and 10*

/_____/

**Section 3
Feedback on the new presentation of allergy and AIT**

1. You have just read the new presentation. Which words would you use to describe it?

*Open-ended question*

1. Which items about **allergies** do you remember?

*Open-ended question*

1. Which of the following items do you remember reading about **allergies**?

*You may give several answers, if applicable*

- Allergy results from a disorder of the immune system
- Allergy is caused by genetic and environmental factors
- Allergy is a real disease that requires medical management
- Allergy is a chronic disease
- Allergy is a progressive disease that can worsen into asthma
- Allergy has a strong impact on personal/professional life

1. How did the new presentation of respiratory allergies and AIT compare with that given by your physician?

In terms of detail: *Please give one answer only*

- - More detailed
  - Less detailed
  - Much the same

In terms of language/wording: *Please give one answer only*

- - Easier to understand
  - More difficult to understand
  - Much the same

1. Which items about **AIT** do you remember?

*Open-ended question*

1. Which of the following items do you remember reading about **AIT**?

*You may give several answers, if applicable*

AIT:

- alleviates symptoms
- is effective on all symptoms
- rebalances the immune system
- induces tolerance to allergens
- has long-term efficacy
- is intended for patients who cannot manage their allergy with symptomatic drugs alone
- is a targeted/tailored treatment
- exists as several modes of administration (shots/injections, drops and tablets)
- reduces the use of symptomatic medications.

1. Is the way AIT works clearly explained in the new presentation?

- Yes
- No

1. To what extent would you say the benefits of AIT are convincing, on a scale from 0 (not convincing at all) to 10 (extremely convincing).

*Please enter a number between 0 and 10*

/_____/

1. Would you say that AIT could meet your needs?

- Yes
- No: please specify:__________________

1. Would you say that this new presentation reassures you about AIT?

- Yes
- No

1. How does this new presentation of AIT compare with that given by your physician?

In terms of detail: *Please give one answer only*

- - More detailed
  - Less detailed
  - Much the same

In terms of language/wording: *Please give one answer only*

- - Easier to understand
  - More difficult to understand
  - Much the same

In terms of convincingness: *Please give one answer only*

- - More convincing
  - Less convincing
  - Much the same

1. Is there anything that your physician told you about AIT that is not included in this new presentation?

- Yes, please specify: ………
- No

1. On the other hand, is there anything included in this new presentation that your physician did NOT tell about AIT?

- Yes

If yes, please specify: *You may give several answers, if applicable*

- - Different forms are available (drops, tablets, shots/injections)
  - Tailored /targeted treatment
  - Long-term efficacy
  - Induces tolerance to allergens
  - Rebalances the immune system
  - Has an effect on all the symptoms
  - Allows a reduction in the use of symptomatic medications
  - Other: please specify:____________________
- Are these items important to you?
  - Yes
  - No
- No

**Section 4
Assessment of willingness to start or restart AIT after the new presentation**

1. Now imagine that your physician had suggested AIT to you after having given the new presentation.

- Would you have felt better informed?
  - Yes
  - No
- Would you have been more inclined to start /resume AIT?
  - Yes
  - No

1. Now that you have viewed the new presentation, do you think that you will start/restart AIT?

- Yes
- No

1. Why?

*Open-ended question*

1. After having viewed the new presentation, how would you evaluate your willingness to start or resume AIT, on a scale from 0 (you absolutely do not want to start or resume AIT) to 10 (you absolutely want to start or resume AIT)?

*Please enter a number between 0 and 10*

/_____/
